# Advances in Therapeutic Monitoring of Lithium in the Management of Bipolar Disorder

**DOI:** 10.3390/s22030736

**Published:** 2022-01-19

**Authors:** Mahsa Sheikh, Meha Qassem, Iasonas F. Triantis, Panicos A. Kyriacou

**Affiliations:** Research Centre for Biomedical Engineering, City University of London, London EC1V 0HB, UK; Meha.Qassem@city.ac.uk (M.Q.); Iasonas.triantis.1@city.ac.uk (I.F.T.); P.Kyriacou@city.ac.uk (P.A.K.)

**Keywords:** lithium monitoring, lithium sensors, therapeutic drug monitoring, bipolar disorder

## Abstract

Since the mid-20th century, lithium continues to be prescribed as a first-line mood stabilizer for the management of bipolar disorder (BD). However, lithium has a very narrow therapeutic index, and it is crucial to carefully monitor lithium plasma levels as concentrations greater than 1.2 mmol/L are potentially toxic and can be fatal. The quantification of lithium in clinical laboratories is performed by atomic absorption spectrometry, flame emission photometry, or conventional ion-selective electrodes. All these techniques are cumbersome and require frequent blood tests with consequent discomfort which results in patients evading treatment. Furthermore, the current techniques for lithium monitoring require highly qualified personnel and expensive equipment; hence, it is crucial to develop low-cost and easy-to-use devices for decentralized monitoring of lithium. The current paper seeks to review the pertinent literature rigorously and critically with a focus on different lithium-monitoring techniques which could lead towards the development of automatic and point-of-care analytical devices for lithium determination.

## 1. Introduction

Lithium was discovered as a therapeutic remedy for psychiatric conditions in the mid-19th century and was reintroduced one century later [1] and it is still the most widely used medication for long-term management of bipolar disorder, where it is administered as a salt in the form of lithium carbonate/cirate/chloride/or sulfate. Bipolar disorder (BD) is a serious life-long disorder, characterized by recurrent episodes of depression and mania [2]. BD is classified, based on the presence of depressive along with manic or hypomanic episodes, into bipolar type I and type II disorder [3]. In bipolar II disorder, depression predominates, and the manic episodes are milder and briefer. This milder and less prolonged form of mania is referred to as hypomania. A person with bipolar I disorder, however, will experience a full manic episode and may or may not experience a major depressive episode [3]. BD affects 2.4% of the world population and is a leading cause of disability worldwide [4]. In its more severe forms, BD is associated with significant impairment of personal and social functioning and high risk of death through suicide as well as poor physical health. Lithium, having the strongest evidence of long-term relapse prevention, is the first-line treatment for both acute and maintenance treatment of BD. Furthermore, lithium is also prescribed for major depressive disorder as an adjunct therapy, as well as a treatment of vascular headaches and neutropenia [5,6]. In addition to its mood-stabilizing properties, remarkable neuroprotective and antiviral properties have also been attributed to lithium, with the use of lithium recently proposed as a potential treatment for Covid-19 [7]. Altogether, it is estimated that up to one million people worldwide take lithium on a daily basis [8].

Despite its global therapeutic use, the benefits of lithium are restricted by its narrow therapeutic index and the incidence of adverse effects [9]. The narrow margin between the safe and potentially toxic doses of lithium has resulted in self-administration of toxic doses accounting for 20–27% of hospitalized poisoning [9], and mortality rates of 9 to 25% reported from lithium toxicity [10]. The therapeutic effect of lithium salts is directly associated with its level in blood serum and there exist differences in individual pharmacokinetic and risk of intoxication [11]. Based on the differences in the excretion rates between individuals, the daily lithium dosage can vary between 10 and 80 mmol, which results in plasma concentrations of 0.4–1.2 mmol/L for effective treatment [12]. Lithium plasma levels greater than 1.2 mmol/L are potentially toxic and can be fatal. Therefore, avoidance of lithium intoxication has been, and continues to be, an important component in lithium treatment, and lithium serum levels must be monitored constantly to ensure its effectiveness and prevent adverse effects [9]. Lithium toxicity is associated with neurotoxic events, hyperthyroidism, hypercalcemia, and other serious conditions [10]. Considering the potential consequences of lithium toxicity, vigilant monitoring should be central in the treatment of BD patients. In the majority of cases, lithium toxicity is preventable with regular monitoring which can significantly reduce the number of toxic episodes in lithium-treated patients [9].

Additionally, treatment non-adherence is a persistent problem in psychiatry, with about 54% of patients not adhering to their prescription [10]. Despite lithium’s proven benefits regarding the prevention of severe affective episodes and suicide, discontinuation of lithium treatment is common amongst bipolar patients with about half of all individuals on lithium medication stopping their treatment at some point, which results in high levels of relapse. Along with psychiatric and physical reasons interfering with lithium treatment, lithium discontinuation has been suggested to be mainly due to its adverse effects which substantially impair the quality of life. Common adverse effects leading to lithium discontinuation are diarrhea, tremor, polyuria/polydipsia/diabetes insipidus, creatinine increase, and weight gain [13]. Therefore, side effects, toxicity burden associated with lithium medication, and the need for regular monitoring via vein puncture are the main reasons for lithium discontinuation and treatment non-adherence [9]. Consequently, the toxicity and treatment non-adherence burden associated with lithium medication has resulted in the widening of the mortality gap between BD patients and the general population. It is hence crucial to develop strategies to improve adherence and prevent unnecessary termination of lithium treatment [13].

Reaching and sustaining the right therapeutic level to avoid toxicity, dose-related adverse effects, and consequently, treatment non-adherence, requires regular therapeutic monitoring of lithium concentrations. In general, peak lithium concentrations in plasma occur two to four hours after an oral dose, with complete absorption occurring at around eight hours. Therefore, it is crucial to monitor lithium levels in serum twelve hours after the last dose [12]. Currently, lithium determination is performed by withdrawing blood samples from the patient by trained personnel, which is invasive and often painful, especially in patients with difficult venous access. The sample is then transported to the central laboratory where blood cells are removed before measurement, a procedure that can take up to 45 min. The quantification of lithium in clinical laboratories is performed by atomic absorption spectrometry (AAS), flame emission photometry (FEP), or conventional ion-selective electrodes (ISEs) [9,14]. These techniques are costly and require highly qualified personnel and elaborate laboratory methods that cannot be translated into point-of-care devices for personal monitoring. Moreover, with the current techniques, the whole analytical process of adjusting the dose after the first administration may have a variable lag time, usually from a few days to weeks [15]. The delay between sample extraction and the analysis of the results restricts the possibility of early detection, correction of problems, and prevention of potential adverse effects.

It is vital for BD patients to have comprehensive information about their pharmacological treatment at the appropriate time as it can substantially improve treatment adherence [13]. Patients’ perception of side effects or apprehension of lithium intoxication, as opposed to the actual presence of side effects, may further contribute to treatment non-adherence [9]. Therefore, there is a crucial need to have quicker and simpler ways to determine lithium levels. Development of a minimally invasive lithium-monitoring method will be a major advance in lithium therapeutic monitoring as it allows patients and non-medically-trained personnel to measure lithium levels and ensure patients are receiving the optimum dose [16]. Moreover, monitoring of drugs with narrow therapeutic range at home using wearable sensors can help reduce the burden on patients and health professionals associated with attendance of clinical settings and facilitate improved therapeutic monitoring [17]. Improved rates of lithium treatment monitoring will majorly enhance the quality of life of bipolar patients and will allow bipolar patients to monitor the drug during their treatment rather than taking single measurements weekly or monthly, which will greatly reduce the risk of adverse effects during the course of their treatment. The pivotal need for the development of low-cost and easy-to-use sensors for decentralized monitoring of lithium has resulted in a great interest in investigating novel lithium-monitoring techniques. Efforts have been made to develop minimally invasive point-of-care lithium-monitoring devices utilizing matrices such as blood, urine, sweat, saliva, and interstitial fluid (ISF). The current paper reviews the literature on investigated technologies for non-invasive monitoring of lithium medication in the treatment of BD.

## 2. Methods

**Figure 1:**
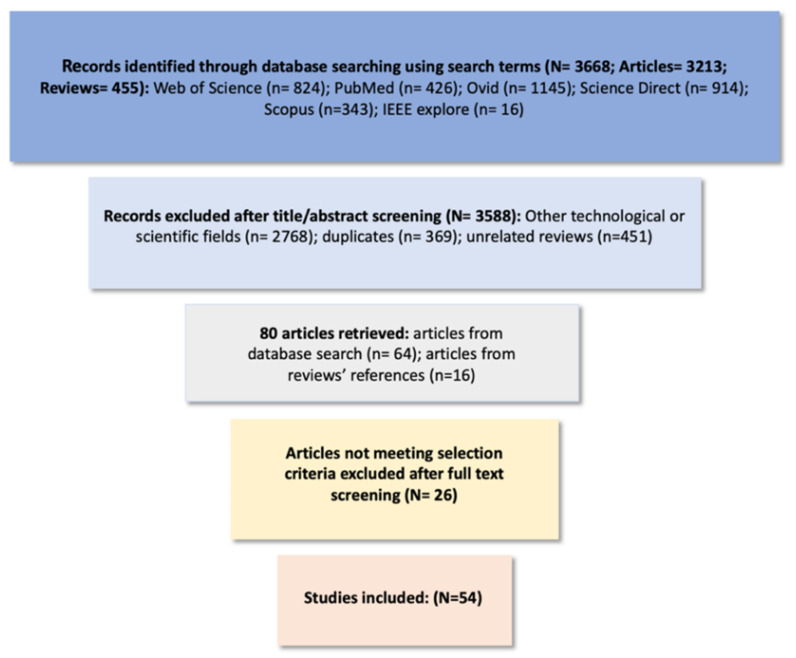
Diagram of the methodology used for the literature review process.

Searches of the literature were conducted in Web of Science, PubMed, Science Direct, Scopus, and Ovid (including journals from Ovid, CityLibrary Journals@Ovid, Allied and Complementary Medicine (AMED), Embase, Global Health, and Ovid MEDLINE). Keywords used in this search included “lithium,” “therapeutic monitoring,” “bipolar disorder,” “lithium monitoring,” and “lithium sensors.” In order to identify any technological advancement in lithium therapeutic monitoring, the studies were included without any time restriction. Database searches yielded 3668 results, of which 455 were review papers. A manual search of all included bibliographies in the relevant review papers was carried out to identify any omitted articles. Studies dedicated to lithium battery domain applications as well as studies published in a non-English language were excluded, and duplicates were removed. From the combinations of the keywords and 16 relevant articles found in review references, 80 articles were identified. Only papers investigating a technological method for monitoring lithium levels in the management of BD were included in this review. Following full-text analysis of the 80 retrieved articles, 54 studies met the inclusion criteria and were included for analysis (Figure 1).

**Figure 2:**
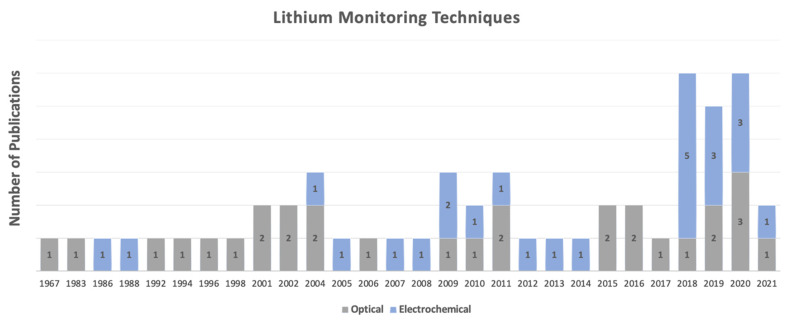
Number of publications investigating lithium therapeutic monitoring systems from 1967 to 2021.

From the 54 studies included in this review, 29 studies employed an optical technique such as atomic absorption spectrometry, flame emission photometry, spectrophotometry, colorimetry, and fluorometry, for lithium therapeutic monitoring in different biological fluids as demonstrated in tables in Section 3 and Section 5.1.3. The remaining 25 studies utilized an electrochemical method including ion-selective electrodes and capillary electrophoresis for detection and quantification of lithium in different matrices, which are summarised in the table in Section 5.2.2. The years of publications analyzing different methodologies for lithium therapeutic monitoring ranged from 1967, initially employing atomic absorption spectrometry, to 2021 (Figure 2). As demonstrated in Figure 2, a great number of publications were produced between the years 2018 and 2020, with a clear increase in the number of studies utilizing electrochemical methods in the more recent years. The significant shift in the number of studies investigating different methodologies in recent years demonstrates the call for more practical and accessible technologies to replace the conventional methods of lithium monitoring.

**Figure 3:**
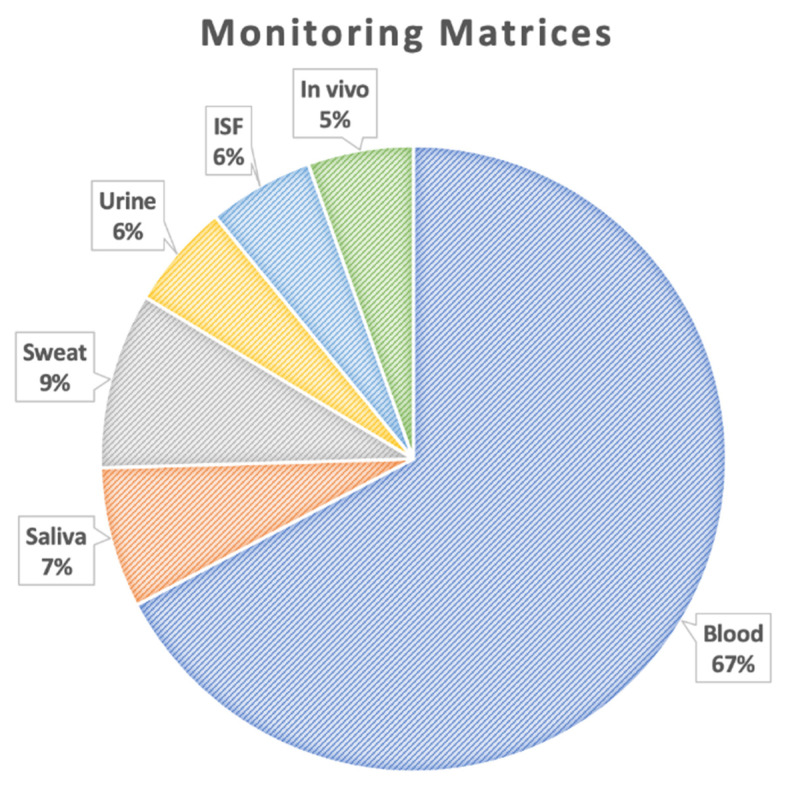
Percentage of studies monitoring lithium in each matrix including blood, saliva, sweat, urine, interstitial fluid (ISF), or in vivo.

In sorting the included studies by the matrix utilized for monitoring lithium therapeutic levels (Figure 3), it is demonstrated that in the majority of publications (67%) lithium was detected and/or quantified in blood. Monitoring lithium levels in other biological fluids including saliva, sweat, ISF, and urine accounted for only 6–9% of the studies, each. In addition, 5% of the studies also proposed in vivo monitoring of lithium levels utilizing different techniques such as clinical scanners for in vivo monitoring of the human brain [18] and blood microsensors [19]. It can be postulated from Figure 3 that there is a need for further research investigating more accessible biofluids as alternatives to blood for lithium therapeutic monitoring. While frequent monitoring of lithium levels in blood is associated with discomfort and consequent treatment non-adherence, alternative matrices might have the potential for minimally invasive and continuous therapeutic drug monitoring (TDM).

## 3. Therapeutic Monitoring of Lithium Levels in Different Biological Fluids

A number of analytical methods have been investigated for lithium measurement in a wide range of media with traditional venous sampling being the “gold standard”. Although this medium allows accurate determination of lithium therapeutic levels, its relative invasiveness, cost, and impracticalities, e.g., need for trained personnel, make it less suitable for frequent or self-monitoring purposes. Therefore, several matrices including sweat, saliva, and dermal interstitial fluid (ISF) have been investigated as potential alternatives to blood for TDM to alleviate the issues related to venous blood sampling and allow minimally invasive monitoring of lithium levels.

The dried blood spots (DBS) sampling, consisting of collecting a blood drop from a finger prick onto a standardized filter paper card, has arisen as an alternative sampling strategy in TDM. The DBS sampling has some advantages in comparison with the standard venous blood collecting, which include its small collection volume, having painless and easy sample collection with minimal training required, and being stable and transportable at ambient temperatures [20]. Dried plasma spots (DPS), representative of serum levels, can also be obtained from a finger prick with the use of membranes that filter erythrocytes [21]. Determination of lithium in dried blood spots (DBS) and dried plasma spots (DPS) by graphite furnace atomic absorption spectrometry (GFAAS) has been investigated [21]. The study showed that lithium was stable in dried samples for twenty days when stored at temperatures up to 42 °C, and that DPS had comparable lithium ion (Li+) concentrations to the ones found in fresh serum. Moreover, it was demonstrated that DBS samples can be used to estimate the Li+ erythrocyte to plasma concentration ratio (LiR), as Li+ erythrocyte level has been suggested to correlate with the occurrence of side effects and therapeutic effectiveness [21]. Nonetheless, dried blood spot analysis is associated with a number of challenges, including sensitivity, reproducibility, and overall accuracy of quantification [22].

**Figure 4:**
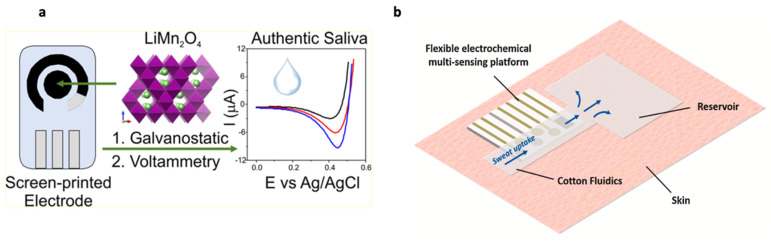
(**a**) LiMn2O4-modified electrodes used for the quantification of lithium ions in saliva (modified with permission from Suherman et al., *ACS Sensors*, 2019 [24]). (**b**) Flexible electrochemical multi-sensing platform used for quantification of lithium concentrations in sweat (modified with permission from Criscuolo et al., *Sensors and Actuators*, 2021 [31]).

Saliva, being a highly accessible biofluid, has also been proposed as a practical matrix for lithium therapeutic monitoring in various studies (Figure 4a) [23,24,25]. The salivary lithium concentrations have been reported to be approximately 2.5 times higher than serum lithium levels, with the effective therapeutic range in saliva being 1.5–2.5 mmol/L, thus demonstrating a significant correlation factor [24,25,26,27]. The elimination of lithium ions from saliva is slower than that from serum, with an elimination half-life of 34.6 h and 24 h for saliva and serum, respectively. This may provide an explanation for the higher concentrations of lithium in saliva [23]. To investigate the correlations between lithium serum and saliva levels and achieve accessible measurements of lithium levels, Serdarevic et al. [23] monitored samples of serum and saliva following two and twelve hours from the last lithium dose using Vitros Analyse 250 dry slide technology and atomic absorption spectrometry (AAS) [23]. The study reported a highly significant correlation between saliva and serum lithium concentrations, with the highest lithium concentration in saliva occurring after two hours of lithium intake [23]. However, it should be noted that saliva has some limitations as the biological matrix for TDM, which relate to drug instability, presence of potential contaminants, and lack of phase II metabolites [28].

A correlation between the level of lithium in sweat and in serum has been demonstrated with the concentration in sweat being roughly three times higher [29]. Therefore, non-invasive therapeutic monitoring of lithium in sweat has been investigated. A microfabricated flexible electrochemical sensor made of a platinum potentiometric sensor with an electrochemically nanostructured solid-contact and a silver reference electrode (RE) has been designed as a wearable headband for remote monitoring of lithium levels in sweat (Figure 4b) [14,29,30,31]. Although sweat was reported to be suitable for non-invasive and decentralized monitoring of lithium, sweat-based TDM suffers from several issues such as possible presence of contamination and concerns regarding drug stability and concentration accuracy with sweat evaporation. Additionally, sample collection requires stimulating sweat glands by different methods such as exercise or thermal heating. Lastly, sweat glands are unevenly distributed; hence, analyte concentration profiles may be location-dependent.

Dermal interstitial fluid (ISF), the fluid bathing the viable tissue of skin, is an accessible and reproducible matrix that is suitable for minimally invasive detection of biomarkers with good correlation with venous blood [8]. A good correlation between the concentration and dynamics of many drugs in ISF and venous blood has been suggested [32]. Therefore, interstitial fluid has previously been proposed as a feasible and stable alternative to blood for TDM, as it has a composition similar to plasma, with the exception of plasma proteins, and sampling ISF is potentially non-invasive and painless [16]. Moreover, absence of clotting factors in ISF makes it suitable for continuous monitoring [16]. A study by Leboulanger et al. [33] demonstrated that a relationship exists between lithium serum concentrations and the lithium extracted by transdermal iontophoresis across the skin. This was achieved by performing reverse transdermal iontophoresis on 23 bipolar or schizoaffective patients to extract lithium across skin by application of an electric current (0.8 mA). Ion chromatography was then used to quantify the extracted lithium and other ions (in the electrode chamber solution), whilst lithium in the blood samples was quantified using ion-selective electrode.

Accordingly, some studies have proposed methodologies for monitoring of lithium concentrations within ISF [33,34]. Hydrogel-forming microneedle(s) (MN) arrays have been investigated for minimally invasive extraction and measurement of lithium in vitro and in vivo [16]. The study suggested MN arrays, composed of tiny projections (50–900 μm in height) attached to a base support, as an alternative approach to ISF sampling. MN arrays were prepared from aqueous combinations of hydrolyzed poly(methyl-vinylether-co-maleic anhydride) and crosslinked by poly(ethyleneglycol), and could absorb ISF upon skin insertion. Following insertion into the skin, MN bypassed the stratum corneum, the skin’s outermost barrier layer, and extracted ISF with little to no pain or bleeding. Therefore, they could rapidly uptake ISF and the containing analyte of interest. Concentrations of lithium in the extracted ISF using MN arrays in both in vivo and in vitro studies were then determined by FAAS [16]. Despite the good accessibility and physiological significance of ISF, it has been challenging to extract a sufficient amount of ISF with high speed. Therefore, the major limitation of ISF for TDM is extracting a reliable amount of analytes for downstream analysis. Utilizing ISF for TDM requires the development of simple and minimally invasive sampling methods that collect more than nanoliter volumes of ISF. Alternatively, development of microneedle sensors that can penetrate the stratum corneum and provide the continuous and real-time monitoring of ISF without the need for extraction is of great interest. Altogether, ISF is an accessible and more reproducible matrix compared to the other biological fluids; hence, it seems to be the most suitable human body fluid for minimally invasive and continuous monitoring of analytes. Lastly, several reports have assessed lithium in urine, as approximately 90% of lithium is eliminated by the renal route [35,36,37]. The studies have utilized colorimetric [35] or capillary electrophoresis [36] techniques, both of which suffer from some limitations, including unsatisfactory accuracy, the need for pretreatment, and wide variations between patients.

Overall, venous blood remains the most utilized medium for therapeutic monitoring of lithium levels. However, blood is not suitable for minimally invasive and continuous monitoring of analytes. Amongst alternative biological fluids, ISF seems to offer a minimally invasive and accessible microenvironment for continuous monitoring of analytes. Moreover, although alternative matrices offer great potential for TDM applications, they have constraints associated with established correlations with blood concentrations, stability, and potential presence of contaminants (Table 1). Therefore, intensive clinical validation studies and reliable correlations linking the measured analyte levels to free blood concentrations are the major prerequisites for alternative matrices. TDM in blood also needs to be advanced to reduce the sample volumes for analysis and minimize the degree of invasiveness.

## 4. Conventional Lithium-Monitoring Techniques

In 1967, the measurement of lithium ion concentration in serum by atomic absorption spectrometry (AAS) was reported by Bowman [38], where atomic absorption measurement of lithium in blood serum was performed using both the conventional dispersion-type monochromator and resonance monochromator [38]. Thereafter, measurement of lithium ion concentration in serum by flame photometry was achieved [39], which unlike AAS, did not require dilution of blood serum. In a study by Doku et al. [39], a flame photometric flow-injection analysis (FIA) method was introduced as an alternative to the traditional batch technique used in flame photometry to reduce the sample volume and achieve faster analysis. The FIA method provided the simultaneous determination of lithium, sodium, and potassium at a rate 10 times faster than the batch method, thereby reducing both sample volume and analysis time [39].

In the 1980s, electrochemical detection methods, including ion-selective electrodes [40,41] and capillary electrophoresis [42] were reported. Several colorimetric [43] and fluorometric [44,45] approaches were also developed in the 2000s [46]. Although some success has been made with these recent techniques, issues exist which relate to interference with other ions present in the sample [47], drifting of electrode-electrolyte interface impedance [48], and need for sample filtration [49]. Therefore, flame emission photometry, atomic absorption spectroscopy, and ion-selective electrodes remain as the routine techniques employed in clinical laboratories for the measurement of lithium levels.

## 5. Advances in Therapeutic Monitoring of Lithium Levels

### 5.1. Optical Methods

The optical sensing of lithium provides various advantages, including rapid sensing, high sensitivity, absence of electrode contact, and effortless maintenance. Chemical ligands such as organic molecules or inorganic materials have been used for optical Li+ detection and/or quantification (Table 2). Lithium measurements can be performed based on absorbance change (colorimetry) or fluorescence emission (fluorometry). A lithium ligand unit and a chromophore/luminophore unit are required for formation of lithium coordination complex, which is directly associated with its detection [50]. Therefore, the optical sensors are designed and fabricated by insertion of a ligand into a detection system such as a membrane or an optical fiber [50]. A number of studies have employed colorimetric reagents such as thoron [51] and quinizarin [52] as optical ligands that are able to form a complex with lithium and result in a shift of its spectrum. Based on their cavity size, crown ethers can also be used as lithium ionophores. Various crown ethers [45] have been synthesized to achieve high selectivity of lithium ionophores by varying the cavity size, conformational flexibility, and different side groups in order to accommodate the metal ion. Due to their lipophilicities, these ionophores can be used to extract Li+ from aqueous solution into organic solvents to achieve spectrophotometric analyses of lithium levels. Therefore, selective lithium extraction towards sodium cations can be achieved based on the cavity size of the crown ether used [50]. Nevertheless, the development of optical sensors utilizing different chemical ligands for selective and efficient lithium monitoring requires achieving high levels of stability and reversibility and short response time.

#### 5.1.1. Spectrophotometry

As aforementioned, lithium can be detected based on chelation-induced absorbance change. In 1983, Trautman et al. [51] achieved the determination of lithium in blood based on the reaction of Li+ with thoron [l-(o-arseno-phenylazo)-2-naphthol-3,&disulphonic acid, sodium salt], which resulted in a bathochromic shift in the spectrum [51]. This chelation-induced absorbance change was measured at 480 nm against a reference reagent. It should be noted that pretreatment procedures were required to remove serum proteins using trichloroacetic acid and to compensate the effect of serum electrolyte by adding a synthetic serum electrolyte to the reagent blank [51]. Another example of detecting lithium based on absorbance change is the application of a synthesized porphyrin, which resulted in a shift of absorption measured at 490 nm after Li+ coordination [53]. The water-soluble porphyrin was synthesized for the spectrophotometric determination of lithium in human serum. Porphyrin did not react with other ions, including sodium and potassium. However, since proteins react with the porphyrin and reduce absorbance, pretreatment procedures were performed to remove the proteins in serum prior to measurement [53]. The use of porphyrin for lithium detection was also investigated by Rumbelow et al. [54] employing a spectrophotometric lithium assay, in which lithium ions were selectively “caged” in a substituted porphyrin compound. Under alkaline reaction conditions, the porphyrin-Li+ coordination resulted in an absorbance decrease which was assessed using a Hitachi 917 analyzer (International Diagnostic Equipment, LLC, Temecula,CA, USA) [54]. The assay was proposed to provide an alternative method for the conventional flame photometer measurements and showed good precision for a range of lithium concentrations from 0.5 to 3 mmol/L. However, this assay required recalibration every 24 h. Additionally, the reagent used for lithium detection was not highly stable and had to be protected from light and CO_2_ absorption to prevent reagent deterioration [54]. Altogether, the molecular ligands used in the majority of optical sensors offer easy tuning of optical properties with rational molecular design. However, lithium detection using optical sensors often suffers from limitations regarding reversibility. This is because after Li+ detection, the lithium sensor is classically non-reusable due to the low reversibility or the non-reversibility of Li+ coordination in the ionophore unit [50]. Therefore, optical properties of inorganic materials or nanocomposites should be more exploited to develop reversible and innovative optical lithium sensors.

The spectroscopic detection of signals can be coupled with different lithium detection techniques to achieve rapid and accurate TDM. For example, Qassem et al. [52] developed a novel method of lithium measurement by combining optical techniques, based on the reaction of Li+ with the chromogenic agent quinizarin, with electrical impendence spectroscopy techniques [52,55,56]. The methodology was tested on blood plasma containing lithium levels in the therapeutic range normally found in bipolar patients. In this study, optical specificity ensured valid qualitative assessment of lithium despite the presence of sodium in high levels and verified the presence of lithium in blood samples. Electrical impedance spectroscopy, on the other hand, offered a high degree of sensitivity to changes in ionic concentration. However, while electrical impedance spectroscopy offers exceptional accuracy in detecting conductivity variations, electrical impedance sensing using non-functionalized electrodes lacks specificity to a single element. Therefore, it was demonstrated that the integration of optical and impedance measurements offering a high degree of selectivity and sensitivity, respectively, could provide an improved method for lithium determination [56,57,58]. Titrimetric analysis has also been coupled with optical spectroscopy to achieve Li+ detection, utilizing an ionophore-based titration reagent [59]. Titrimetric assay is a method of measurement with high precision and is only limited by the challenge of using the right chelators to achieve sufficient selectivity for the target ion against interfering ones [59]. In this study, following complexation of the lithium with Li+ titration reagent in dichloromethane (CH_2_Cl_2_), the optical signals were measured with a UV-visible absorption spectrometer to acquire the absorption spectra. However, the results showed that the Li+-selective reagent could not tolerate high concentrations of Na+ because of the limited selectivity, and was not suitable for Li+ titration in serum [59].

In the optical detection of heavy metals, the development of optical chemical sensors (optodes) for quick and inexpensive sensing of analytes is of great interest. Typically, optodes employ a plasticized polymeric membrane, and are based on the reversible mass transfer of analyte from the sample into the bulk of the sensing layer [60]. Therefore, various ionophores and appropriate lipophilic pH indicator dyes can be introduced into the membranes to design optical cation-sensing systems [60]. Albero et al. incorporated a bulk optode for flow injection–spectrophotometric determination of lithium in pharmaceuticals and human saliva [60]. The flow-through optode membrane was based on an ionophore which formed a complex with Li+ acting as a neutral ligand, and a chromoionophore which interacted with the reference ion (H+) and changed optical properties upon protonation (the indicator was green in its acidic form and yellow in its basic form). A lipophilic cation-exchanger was also involved in the response mechanism and determined the diffuse reflectance spectra of the optode membrane and the sensor response [60]. While the sensor showed good sensitivity and linear response characteristics, selectivity remained an issue. Another drawback of optodes is the short lifetime of materials used in the fabrication membranes, which can reduce the long-term stability of the sensor.

Colorimetry, including measurement of the wavelength and the intensity of electromagnetic radiation in the visible region of the spectrum, is extensively used for identification and determination of concentrations of substances that absorb light [61]. Colorimetric determination of lithium levels has been investigated by several studies [43,46,52,62]. Colorimetric determination of lithium using a dry slide-based method (from Kodak Ektachem analyzer) was investigated in 1994 by Gorham et al. [62]. The slide incorporated the binding of Li+ to a crown-ether azo dye which resulted in a shift in the peak absorption of the dye from ∼400 to ∼600 nm. The colorimetric shift was then detected quantitatively and correlated with the concentration of Li+ [62]. However, Na+ and hemoglobin demonstrated interference with Li+ determination. Therefore, the impact of these interfering substances must be investigated in clinical management and at different concentrations present in the sample [62]. Zhang et al. also designed and synthesized a macrocyclic Sm(III) complex which served as a colorimetric lithium ligand and selectively recognized Li+ [63]. Following addition of Li+ to the colorimetric ligand in a solution of methanol and water, an absorption band at 301 nm and the broad shoulder peak in the range of 410–475 nm were observed. It also caused a significant colorimetric response from light green to yellow green [63]. Having different photophysical properties in the presence of Li+ against other alkali metal ions, the ligand showed a selective and rapid response to Li+ with a detection limit of 1.147 mmol/L [63]. Lastly, Iwai et al. developed the lithium assay kit LS, which utilized a conventional microplate reader to perform post-mortem analyses of lithium in whole blood and urine samples collected at autopsy [35]. In this colorimetric assay, lithium present in the sample yielded a magenta-colored complex when treated with polyfluoroporphyrin as the chromogen. Thereafter, the optical density was measured at 550 nm with a reference wavelength of 600 nm using the microplate reader [35]. To achieve a calibration curve with good linearity for lithium in blood, the study had to perform pretreatment procedures. This is while good linearity was achieved for urine samples without any pretreatments. Furthermore, unlike samples in the higher concentration range (2–20 μg/mL), the study could not achieve satisfactory accuracy and precision values for lower concentrations of lithium (0.5–1 μg/mL) [35]. Moreover, a major limitation in the development of organic chromophores for practical Li+ detection is their lack of solubility in aqueous media. Accordingly, Obare et al. employed the aggregation-induced color changes of gold nanoparticles in aqueous solutions as an optical method for lithium detection [43]. In this study, gold nanoparticles were used as colorimetric indicators and were functionalized with 1, 10-phenanthroline ligand that selectively binds to Li+. The ligand-metal complex resulted in the aggregation of Au nanoparticles, the degree of which depended on the Li+ concentration. The aggregation of Au nanoparticles then caused a shift in the extinction spectrum which was monitored at 620 nm, with a concomitant color change due to mutually induced dipoles [43]. Nevertheless, this study also suffered from interference with other ions present in the sample.

**Figure 5:**
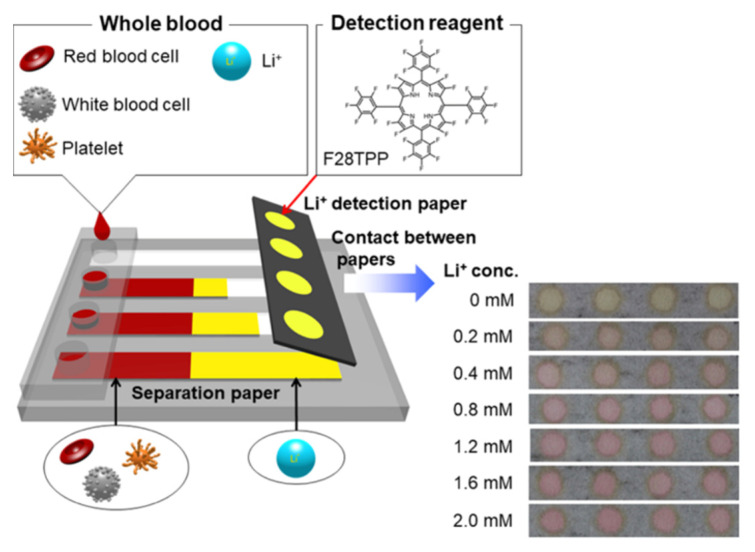
Paper-based device for colorimetric determination of lithium ions in human whole blood (modified with permission from Komatsu et al., *ACS Sensors*, 2020 [46]).

Colorimetric determination of lithium in blood samples has also been performed using Dimension Xpand analyzer, which provided a fully automated and quantitative assay based on the change in absorbance at 540 nm [64]. The colorimetric method showed no interference with other substances in the sample including Na, K, Ca, and Mg, and a shorter turnaround time, of around 45 min [64]. While the study reported a shorter turnaround time compared to the conventional methods such as flame photometry, facile monitoring of lithium levels requires more rapid detection methods. An example of point-of-care testing for lithium monitoring is a colorimetric paper-based device developed by Komatsu et al. which consists of a blood cell separation unit and a colorimetric detection unit [46]. In this system, the blood cells were separated from whole blood and then the lithium-ion detection unit pulled up the plasma from the separation unit (Figure 5) [46]. Thereafter, the reaction of the F28 tetraphenylporphyrin (F28TPP) detection reagent and the Li+ cations yielded a magenta diagnostic color. Ultimately, the blood ion concentration was determined using image analysis by taking a photograph of the detection unit [46]. Although the paper-based device allowed point-of-care testing of whole blood, the colorimetric determination of lithium levels was performed by analysis of the images obtained using a digital camera. Therefore, the accuracy of the measurement might be affected by the resolution of the camera. Fixed shooting conditions as well as machine learning image analysis techniques should be employed in order to increase the accuracy and reliability of the measurements. In conclusion, different chromogenic agents have been introduced for selective detection of lithium ions. However, most of these studies have employed conventional analyzers and spectrophotometers to detect the colorimetric absorption peaks caused by the reaction of lithium ion with the chromogenic agent. Therefore, development of handheld analyzers which provide rapid and facile colorimetric detection of lithium levels is highly required.

#### 5.1.2. Fluorometry

Sensing of analytes using luminescent sensors can be observed via changes in emission intensities, wavelengths, and lifetimes [45]. The detection and quantification of lithium has been achieved based on chelation-enhanced luminescence using ligands such as 1–10 phenanthroline [65]. 1–10 phenanthroline forms a [2:1] coordination complex in the presence of lithium cations and allows the detection of Li+ by increasing the luminescence intensity. Initially, Hitarani et al. [65] demonstrated the selectivity of 1, 10 phenanthroline derivatives for Li+. Consequent to complexation with Li+, these molecules exhibited fluorescence enhancement in the ultraviolet spectrum upon excitation at 298 nm [44]. Later, Obare et al. modified 1, 10-phenanthroline to increase its aromatic nature to benzodipyrido [3,2-a:2′3′-c] phenazine (BDPPZ, 3) coupled with butyl substituents. The synthesized Li+-selective ligand showed changes in emission color upon Li+ binding. The ligand was investigated when dissolved in tetrahydrofuran (THF) and ethanol, emitting in the green (λmax = 538 nm) and yellow (λmax = 553 nm) region of the spectrum, respectively [44].

In 2002, the first fluorescent PET Li+ chemosensor was designed by Gunnlaugsson et al. based on diaza-9-crown-3 as the fluorophore-spacer-receptor [45]. The use of diaza-9-crown-3 as the Li+ receptor showed a reduced Na+ affinity compared to larger crown ethers such as 12-crown-4, which can lead to Na+ interference [45]. Later, in 2011, Kim et al. developed the prototype of a portable device for non-invasive measurement of lithium in saliva [25]. The device contained a photo detector for spectrofluorometric sensing of lithium based on its reaction with 1,4-dihydroxyanthraquinone (quinizarin) and the fluorescence was measured at 620 nm with an excitation wavelength of 590 nm [25]. Overall, the main challenge associated with the employment of fluorophores for lithium detection is their short emission lifetime, as the lifetime of the ligand must be sufficient for correct detection. Furthermore, to obtain efficient lithium coordination as well as selectivity towards competitive alkali cations, balance between the size and the rigidity of the ionophore unit must be achieved [66].

#### 5.1.3. In Vivo Optical Methods

**Figure 6:**
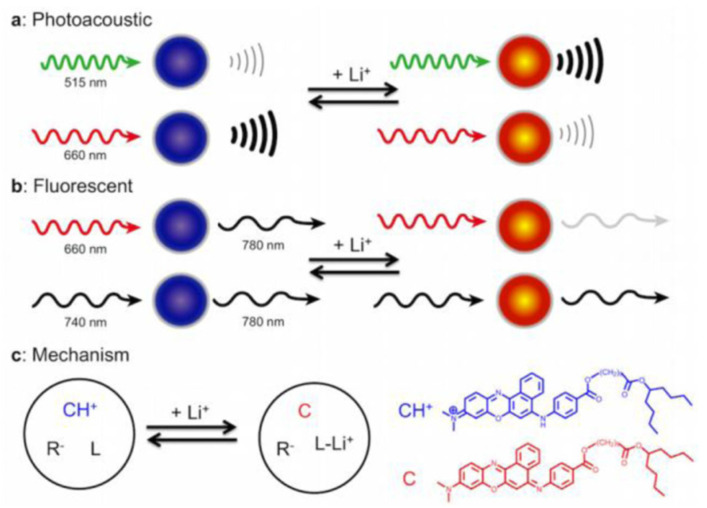
Photoacoustic (**a**) and fluorescent (**b**) imaging techniques were employed to monitor nanosensors used for detecting lithium. In photoacoustic monitoring, two wavelengths were used to interrogate the chromoionophore embedded in the sensors, and the photoacoustic waves from each wavelength changed as lithium concentration varied. In fluorescent imaging, a near-IR fluorophore was added to the sensors and the intensity of FRET from the chromoionophore to the near-IR dye changed with different lithium concentrations. (**c**) The fundamental mechanism of the lithium response was lithium extraction by an ionophore (L) into the core of the nanosensor, which deprotonated a chromoionophore (CH^+^), changing the optical properties of the nanosensor. An additive (R) balanced the charge inside the sensor (modified with permission from Cash et al., *ACS Nano*, 2015 [67]).

Lithium stabilizes mood through complex mechanisms in the brain. However, it has been suggested that serum levels are a poor indicator of the brain lithium concentrations. Therefore, Smith et al. investigated the use of magnetic resonance spectroscopy to achieve in vivo measurement of lithium in a healthy human brain using a 3T clinical scanner [18]. The spectroscopic imaging technique yielded a mean brain lithium concentration of 0.71 ± 0.1 mmol/L, with no significant difference between grey and white matter. The study also calculated a mean brain/serum ratio of 0.78 ± 0.26 with the brain Li concentration reported as ~ 80% of serum levels in healthy subjects [18]. However, the imaging technique suffered from low resolution which reduces the accuracy of lithium measurements. The in vivo monitoring of serum lithium levels without the need for blood sampling has also been investigated [67]. Cash et al. developed lithium-sensitive optical nanosensors for photoacoustic measurements to achieve in vivo monitoring of lithium [67]. Synthetic nanosensors could measure physiologic parameters with photoacoustic contrast, and hence were used to continuously track lithium levels (Figure 6) [67]. The designed nanosensors responded to lithium concentrations by producing a ratiometric photoacoustic index and a ratiometric fluorescent index. However, these multiwavelength indexes suffered from biases resulting from differential tissue attenuation at the two wavelengths. Furthermore, in the development of nanosensors, achieving high spatial resolution as well as optimum penetration depth remains challenging. Moreover, Di et al. utilized erythrocyte-camouflaged microsensors to achieve “red blood cell mimicry” [19]. The developed florescence-based microsensor circulated directly in the bloodstream with long circulation times of up to 2 weeks and provided frequent monitoring of sodium levels. The fluorescence activity of the sensors was detected and quantified by an external optical reader, and the measurement was correlated with a calibration curve to measure levels of sodium [19]. As lithium toxicity results in decreased sodium reabsorption and depletion in blood, the study sought to monitor sodium blood levels to achieve monitoring of lithium and downstream targets [19]. It should be noted that the developed microsensor was only tested in vitro, hence requires overcoming challenges of tissue scattering, stability in biological environment, reversibility, and/or limits of detection in order to be translatable from in vitro to in vivo. Overall, in vivo imaging and monitoring for continuous determination of lithium levels without the need for blood sampling can facilitate personalized medicine where drug dosing is tailored to patients to improve treatment response and minimize adverse side effects. Nonetheless, achieving in vivo monitoring of lithium levels with high levels of accuracy remains challenging. Reversibility of the sensor response, low resolution and imaging depth, and short elimination half-life of the nanoparticles are amongst the challenges that need to be overcome to achieve accurate monitoring of lithium levels in vivo [19,67].

**Table 2:** Studies investigating optical techniques for lithium therapeutic monitoring.

Reference	Type	Sensing Platform	Testing Matrix	Lithium Ligand/Detection Method
Cash et al. [67]	Optical	lithium-sensitive (optical) nanosensors	In vivo monitoring	Photoacoustic imaging
Di et al. [19]	Optical	Erythrocyte-camouflaged florescence-based microsensor	In vivo monitoring	Diffuse in vivo flow cytometry
Smith et al. [18]	Optical	3 T clinical scanner	In vivo monitoring, human brain	Spectroscopic imaging
Albero et al. [60]	Spectrophotometric	Flow-through spectrophotometric bulk optode	Saliva and pharmaceuticals	Ionophore-based poly(vinyl) chloride membrane
Rumbelow et al. [54]	Spectrophotometric	Hitachi 917 analyzer	Serum	Substituted porphyrin compound
Tabata et al. [53]	Spectrophotometric	Shimadzu UV-2100 and Jasco Ubest spectrophotometers	Serum	Porphyrin (octabromoporphyrin)
Trautman et al. [51]	Spectrophotometric	Varian Superscan 3 ultraviolet-visible double-beam spectrophotometer	Blood	Thoron [l-(o-arseno- phenylazo)-2-naphthol-3,&disulphonic acid, sodium salt]
Zhai et al. [59]	Spectrophotometric	UV-visible absorption spectrometer	Serum	Titrimetric detection based on complexation of the lithium with Li titration reagent in dichloromethane (CH_2_Cl_2_)
Komatsu et al. [46]	Optical, colorimetric	Colorimetric paper-based device consisting of a blood cell separation unit and a colorimetric detection unit	Whole blood	F28 tetraphenylporphyrin (F28TPP) was used as the detection reagent
Gorham et al. [62]	Optical, colorimetric	Dry slide-based serum lithium assay	Blood	Absorbance change based on binding of Li+ to a crown-ether azo dye
Iwai et al. [35]	Optical, colorimetric	Lithium assay kit LS coupled with microplate reader	Whole blood and urine	Colorimetric response based on binding of Li+ and polyfluoroporphyrin as chromogen
Gruson et al. [64]	Optical, colorimetric	Dimension Xpand analyzer	Blood	Absorbance change based on the formation of a noncovalent binary complex between Li and 7- nitro-2,12-dicarboxyl-16, 17dihydro-5H, 15H-dibenzo wb,ix (1), 11, (4, 5, 7, 8) dioxatetraaza-cyclotetra-decine in an alkaline mixture
Qassem et al. [52,55,56]	Optical, colorimetric	Optical and electrical impedance spectroscopy	Blood	Combination of optical and electrical impedance spectroscopy, optical detection based on the reaction between Li and quinizarin
Zhang et al. [63]	Optical, colorimetric	Spectra detected on UV-Vis spectrophotometer	Methanol and water solution	Absorbance change and a colorimetric response based on macrocyclic Sm(III) complex serving as a colorimetric ligand for Li+
Obare et al. [43,44]	Optical, colorimetric	Gold nanoparticles	Tested in aqueous solution	Absorbance change and colorimetric response based on binding of Li+ with 1, 10-phenanthroline ligand
Gunnlaugsson et al. [45]	Optical, fluorometric	Fluorescent PET Li+ chemosensor	Tested in queous solution	Diaza-9-crown-3 as the Li+ receptor
Kim et al. [25]	Optical, fluorometric	UV-Vis spectrophotometer	Saliva	1,4-dihydroxyanthraquinone (quinizarin)

### 5.2. Electrochemical Methods

#### 5.2.1. Ion-Selective Electrodes (ISEs)

In the late 1980s, ion-selective electrodes (ISEs) were developed for lithium measurement [68]. Since ion-selective electrodes often suffer from lack of selectivity and sensitivity towards interferents present within the measurement media, different membrane structures and active membrane components have been investigated to provide higher affinity to lithium ions. Therefore, a variety of membranes including polymeric solvent membranes with inner filling solution [40] and solid contact polymeric membranes without inner filling solution [69] have been utilized to construct lithium-selective electrodes (Table 3) [49].

Several studies have attempted to develop conductive polymer sensors for monitoring lithium levels [49,70]. Conductive polymers (CPs) and nanostructures are amongst commercial solid-contact ion-selective electrodes (SC-ISEs). Nanostructured materials including carbon and noble metal nanostructures offer several advantages over CPs which include the possibility to achieve high conductivity, the absence of possible side-reactions, and the insensitivity to pH and light [71]. These SC-ISEs utilize the electrical double layer that is formed at the membrane/electrode interface for ion-to-electron transduction. In this system, the formation of an asymmetric capacitor is achieved by accumulation of ions on one side of the interface which attracts electrons or holes on the other side [30]. By virtue of their affinities to Li+, different ionophores (e.g., amide-type ionophores) have been incorporated into ion-selective membranes or electrodes to achieve ionophore-based sensors [71].

Electrical impedance spectroscopy can also be used for the detection of lithium levels without the need for specific ion-selective membranes or addition of lithium-binding dyes [55]. Tetrapolar electrical impedance spectroscopy (TEIS) has been investigated for the detection of lithium levels by applying an ac current via a pair of electrodes and recording the induced difference in potential via a separate pair [55]. TEIS exhibited lower sensitivity to the electrode-electrolyte interface than bipolar configurations and was shown not to require sample filtration, thus reducing user and sensor errors, making the measurement less elaborate, and reducing the processing time. Furthermore, the proposed TEIS method offered very good sensitivity to lithium changes [55]. However, while TEIS provides accurate detection of lithium variations, it suffers from lack of selectivity to lithium. Therefore, in the study by Qassem et al. [56], electrical impedance spectroscopy was combined with optical methods to achieve a high degree of both selectivity and sensitivity to lithium variations.

##### Potentiometric

In 1986, Metzger et al. investigated an electrode based on PVC membrane containing N,N-dlcyciohexyi-N′,N′-diiso- butyCclscyclohexane-l,2dicarboxamlde (ETH 1810) [40]. ETH 1810 was synthesized and tested in different liquid membranes to evaluate its potential in the potentiometric determination of Li+ in blood serum. Although ion-selective liquid membrane electrodes showed high response times, the membrane showed constraints associated with the Li+/Na+ selectivity [40]. Later, in 1988, Bertholf et al. utilized an ion-selective electrode for the determination of lithium based on the interaction of lithium with an ionophore contained in a sodium-sensitive polyvinyl chloride (PVC) membrane using the Du Pont Na/K/Li analyzer. However, the results showed that with respect to precision and linear range, the routine flame photometric method still outperformed the proposed method [41].

Aniline and its derivatives have been extensively studied as conducting polymers due to their chemical stability, ease of polymerization and doping, as well as low cost [70]. Lindino et al. reported the use of a gold electrode modified with poly(o-methoxyaniline), which is a derivative of aniline polymers, as the conducting polymer to respond to lithium ions in the concentration range of 1 × 10^−5^ to 1 × 10^−4^ mol L^−1^ [70]. The electrode modified with the conducting polymer showed a potentiometric response to Li+ concentration [70]. The results suggested that the performance of the modified electrode was comparable to the standard atomic emission spectrophotometry method. However, it should be noted that the potentiometric response of the modified electrode showed a tendency to decline with time due to the insertion of lithium ions into the polymer structure which affected the electrochemical properties of poly(o-methoxyaniline) [70]. To resolve this issue, the paper suggested the use of flow injection analysis in order to reduce the contact time between the polymer and lithium ions given that the analysis process is dynamic [70]. In this regard, Coldur et al. have developed a micro-sized solvent polyvinyl chloride (PVC) membrane Li+-selective electrode coupled with a potentiometric flow injection system [49,69,72]. The proposed electrode was applied for the potentiometric determination of lithium ions in human serum samples and showed results which were comparable to those of standard measurements [49]. However, the obtained selectivity for Na+ in this study was not adequate for reliable Li+ determination in serum samples. Therefore, it has been suggested that utilization of an ISE array comprising Na+ and K+ selective electrodes in addition to the Li+ electrode might be the solution to the limitation associated with interferents found in serum [49].

**Figure 7:**
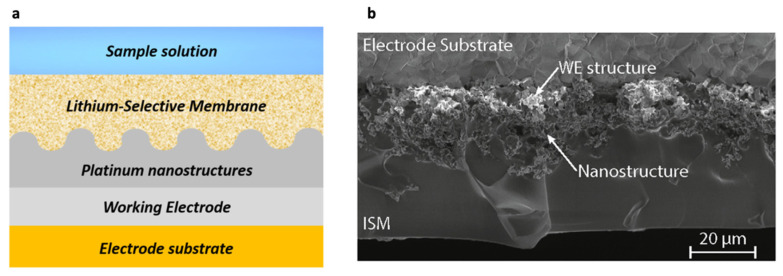
(**a**) The structure of the Li+-ISE-based sensing electrode on platinum nanostructured solid-contacts. (**b**) SEM cross-sectional view of a Li^+^ ISE with platinum nanoflowers as SC (modified with permission from Criscuolo et al., *Analytica Chimica Acta*, 2018 [30]).

All-solid-state ion-selective electrodes (ISEs) with noble metal nanostructured layers as ion-to-electron transducers have been investigated by Criscuolo et al. [30]. The study employed nanostructured gold and platinum contacts that allowed the increase of solid-contact capacitance by one and two orders of magnitude, respectively. The electrochemical measurements showed reduced charge transfer resistance, large capacitance, and good analytical performance [30]. Later, Criscuolo et al. developed a wearable multi-electrode platform based on electrodeposited platinum nanostructures for simultaneous sensing of analytes in sweat (Figure 4b). The electrochemical potentiometric sensor based on all-solid-state ion-selective electrodes (ISEs) was fabricated by depositing an ion-selective membrane (ISM) cocktail containing poly(vinyl chloride) and Li ionophore VI (6,6-Dibenzyl-1,4,8-11-tetraoxacyclotetradecane) on the electrodes (Figure 7) [14,30,31]. The multi-sensing platform contained a temperature sensor and a stable reference electrode (RE) with an ionic-liquid junction and was designed for different applications including Li+ TDM. The platform was tested in water, artificial sweat, and human volunteers for Na+ and K+, but is yet to be tested in human volunteers for Li+ [31]. Similar to [30], Hanitra et al. also investigated screen-printed electrodes which were functionalized by drop-casting a membrane cocktail based on poly(vinyl chloride) and Li ionophore VI to achieve lithium ion measurement [73]. The study included an electrochemical sensing platform which employed potentiometry for Li+ sensing as well as amperometry for lactate detection in sweat. The hardware also combined readout circuitry for amperometric and open circuit potential (OCP) measurements, and a wireless communication was ensured by a Bluetooth low energy (BLE) module [73]. The platform performing both amperometric and potentiometric measurements was then integrated in commercially available armband cases to provide a wearable system for detection of lactate and lithium [73]. Thereafter, Hanitra et al. developed a multiplexed four-channel readout interface which supported amperometric, voltammetric, and potentiometric measurements, and was designed for electrochemical sensing of analytes. The electrochemical sensing of lithium was particularly achieved by measuring the open circuit potential (OCP) between the ISE and a Ag/AgCl double junction electrode [74]. Altogether, employing solid contacts as sensing electrodes requires efficient nanostructuration to improve potential response and stability. Furthermore, the proper conditioning procedure needs to be exploited to ensure high accuracy levels.

**Figure 8:**
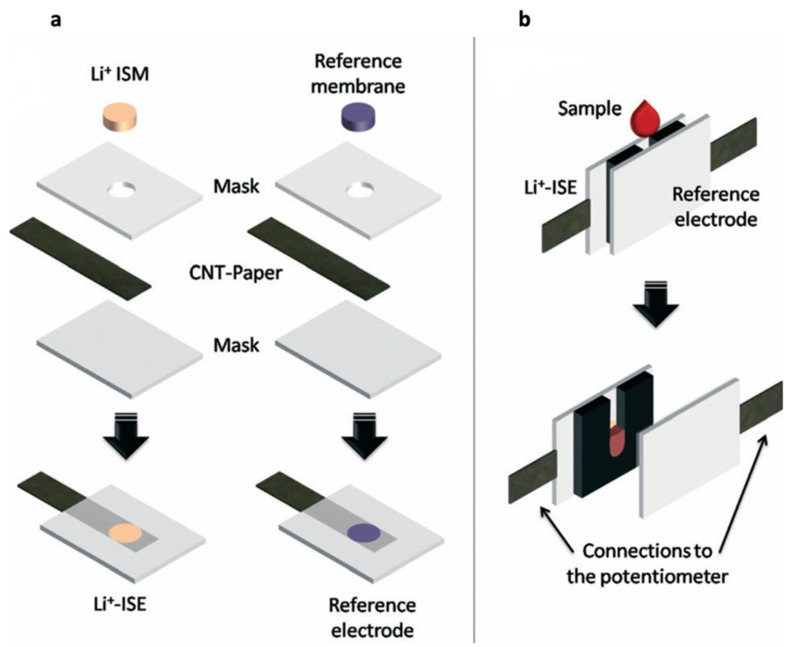
Paper-based potentiometric cell for decentralized monitoring of lithium levels in whole blood. (**a**) A scheme of the paper electrodes. (**b**) A scheme of the completed measuring setup (modified with permission from Novell et al., *Lab on a Chip*, 2014 [15]).

Novell et al. reported the monitoring of lithium levels in blood, in decentralized settings using a potentiometric cell fully made with filter paper (Figure 8) [15]. Solid-state ion-selective electrodes and a reference electrode were built using carbon nanotube ink to create a conductive path and suitable polymeric membrane. The paper-based potentiometric cell was suggested to accurately predict lithium levels in whole blood and serum samples [15]. Nonetheless, decentralized monitoring of lithium levels with the proposed device requires achieving high levels of reproducibility as well as optimization of the cell volume and miniaturization of the electrodes. Ultimately, a flexible cotton-based lithium sensor was coupled with a carbon-fiber-based reference electrode to obtain a fiber-based potentiometric cell for non-invasive therapeutic lithium monitoring in dermal interstitial fluid [75]. The extraction of lithium from under the porcine skin was performed using fiber-based reverse iontophoresis (RI) electrodes which are used for the extraction of ions and non-charged species. In reverse iontophoresis, a small current is applied between two electrodes on the skin which causes the ions in the subdermal layer to flow across the skin to the surface where the electrode of opposite charge is located. Following the extraction of ISF, lithium determination was performed using the miniaturized fiber-based potentiometric sensor by measuring the difference in potential between two electrodes [75]. The cotton-fiber-based lithium sensor was fabricated by dipping a cotton thread in SWCNT ink and lithium membrane solution, and the silver-coated carbon-fiber-based reference electrode was fabricated by dipping carbon fiber in Ag/AgCl and a reference membrane solution (RM) [17,75]. The fiber-based sensor allowed the determination of Li+ concentration change in simulated interstitial fluid. However, the silver ink used in the CNF-Ref sensor showed cytotoxicity and there also remained challenges with sensitive detection of lithium therapeutic levels.

##### Voltammetric

An electrochemical (EC) sensor based on the gradual decrease in the voltammetric current signal of the redox probe indicating the increase of lithium at the sensor surface has been investigated [76]. The electrochemical screen-printed sensor strips were functionalized using 14-crown-4 ether (6,6′-dibenzyl- 14-crown-4 ether)-based ionophore for highly selective monitoring of serum lithium [76]. The ionophore solution was drop casted over the self-assembled monolayer-immobilized gold surface, and the modified screen-printed lithium sensor was connected to the electrochemical analyzer used for recording the current signal [76]. The EC lithium sensor was also fabricated by incubating the strips with 10 μL serum sample spiked with standard lithium solution and measurements were made prior to and after the introduction of the analyte onto the sensor surface. The Li+ sensor electrode was not responsive to the presence of other ions including K+, Na+, and Ca+2 in the serum which indicated the suitability of the proposed sensor strip for serum lithium monitoring. However, further studies are required to model a standard inhibition curve for determining lithium variations for point-of-care clinical applications [76]. Suherman et al. also proposed a lithium-sensing methodology based on the galvanostatic delithiation of lithium manganese oxide (LMO) followed by linear stripping voltammetry (LSV), which allowed the detection of the reinsertion of Li+ (lithiation) in the analyte [24]. Lithium manganese oxide (LMO) has high selectivity for Li+, hence it can be used as an electrode surface modifier for lithium detection. Therefore, lithium manganese oxide (LiMn2O4)-modified glassy carbon electrodes (LMO-GCEs) and screen-printed electrodes (LMO-SPEs) were investigated for the detection of lithium ions in human saliva (Figure 4a). Herein, the use of solids as a selective approach for lithium detection was compared to the use of Li+ ionophore-based membranes. The paper suggested that the use of solids can provide good selectivity with lower cost and synthetic requirements and may be easily screen printed [24]. The results demonstrated that screen-printed electrodes give a clearer response compared to the GCE, with the absolute peak current values increasing linearly with the increasing concentration of Li+-spiked authentic human saliva [24]. However, further optimization of the concentration of the ionophore at electrode surface is required to ensure the gradual decrease of EC signal with increasing metal ion concentration.

#### 5.2.2. Capillary Electrophoresis

**Figure 9:**
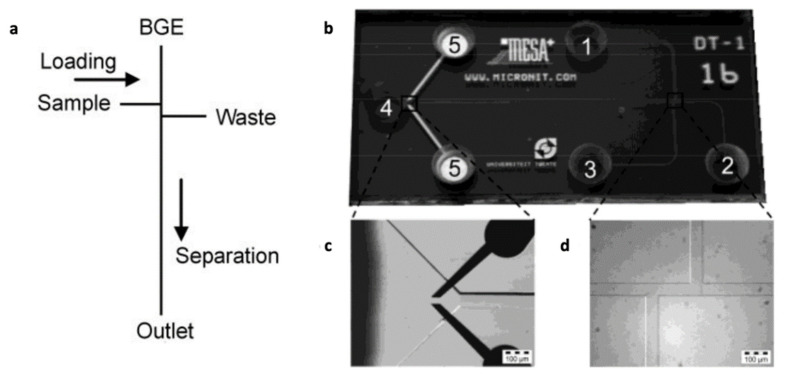
(**a**) Schematic representation of the channel layout of the CE chip with double-T injection geometry (modified with permission from Vrouwe et al., *Electrophoresis*, 2005 [77]). (**b**) Photograph of the microchip: 1, sample compartment; 2, BGE compartment; 3, waste compartment; 4, outlet compartment; 5, detection electrodes. Close-ups of the end of the channel with (**c**) the conductivity detection electrodes and (**d**) double-T injector (modified with permission from Vrouwe et al., *Electrophoresis*, 2004 [42]).

Capillary electrophoresis, an analytical technique specially adapted for the separation of ions, offers great potential for separation and quantification of lithium and other ions in biological fluids (Table 3) [36]. Capillary electrophoresis is suggested to allow simultaneous measurement of multiple ions with minimal interference and without the need for selective electrodes [42]. In microchip capillary electrophoresis, the typical channel layout includes a heart-cutting configuration of two connecting channels (Figure 9a) [77]. The first channel, referred to as sample-loading channel, leads from the sample compartment and transports the sample to the channel intersection. Thereafter, the sample plug is dispensed into the second channel, referred to as the separation channel, which leads from the background electrolyte (BGE) compartment to the outlet for the capillary zone electrophoresis (CZE) separation [77]. The electrokinetic transport of the sample towards the intersection is based on a combination of electrophoretic migration of analytes and the electroosmotic flow (EOF). As the ions migrate from the sample compartment into the loading channel, zones are formed based on the electrophoretic mobility of the ions. Thereby, in a given time period, species with high electrophoretic mobility are able to fill a greater length of the loading channel than species with lower mobility [77].

Indirect UV-absorption, amperometric, and conductometric methods are typically employed for the detection of small ions in CE. Indirect UV-absorption detection lacks sensitivity and amperometric detection requires alignment of the separation channel and sensing electrodes, which must be accomplished either by using a microscope and micro-manipulators or by embedding electrodes into the chip during manufacture [37]. Since all charge species separated in an electrophoretic system can be easily detected, conductivity detection is widely used in microchip analyses [37]. Microchip capillary electrophoresis (CE) has been investigated for point-of-care testing of lithium in whole blood, where the blood components are separated by capillary electrophoresis and lithium is detected by conductimetry [78]. This system combined a glass capillary as a sample collector for blood from a finger stick with a microchip capillary electrophoresis (CE) to provide rapid testing of electrolytes in serum or whole blood [78]. Vrouwe et al. also reported the direct measurement of lithium ions in whole blood, utilizing capillary zone electrophoresis on a microchip based on conductivity detection (Figure 9b) [42]. The microchip CE used in this study worked based on the fact that blood cells have relatively low electrophoretic mobility compared to the alkali metals. This allowed the cell-free sample plugs to be formed in the double-T of a typical microchip CE device [42]. To investigate the behavior of the cells under different electroosmotic flow conditions, the study employed both bare glass chips and chips coated with polyacrylamide. It was demonstrated that coated devices gave reproducible electropherograms, while proteins quickly contaminated the untreated chip surfaces. Furthermore, coated chips had higher efficiency due to the reduction of the electroosmotic flow (EOF), which resulted in improved separation of the ions present in the sample [42]. The main challenge of this technique was the sensitivity of the detector to lithium compared to that of sodium [42]. To overcome this limitation, Vrouwe et al. used sodium as the internal standard. This method resulted in improved quantification of lithium concentration with a detection limit of 0.1 mmol/L in blood plasma [77].

Furthermore, an electrophoresis-based lab-on-a-chip device for the measurement of lithium concentration in blood was introduced by Floris et al. [79]. The device employed disposable prefilled microfluidic chips with closed electrode reservoirs and a single sample opening. The chips were inserted into a hand-held analyzer and the quantification of lithium was achieved by conductivity detection after separation from other blood ions [79]. While the developed device has been tested in several psychiatric wards and clinical laboratories, it requires further improvements for accurate and precise measurement of small concentrations. More recently, Jamal et al. utilized capillary zone electrophoresis for monitoring lithium concentrations in serum, urines (biological fluids), and dialysates (non-biological fluid) of a patient hospitalized with acute lithium poisoning [36]. The study showed a strong correlation between the results from capillary zone electrophoresis (CZE) and flame photometry measurements. Herein, CZE was suggested to provide the quantification of lithium in the therapeutic concentration range as well as in the context of acute intoxication for different types of biological fluids [36]. However, the method demonstrated limits of quantification of 0.15 mmol/L and 0.07 mmol/L for serum and other fluids, respectively [36]. Overall, the major challenge associated with the determination of lithium concentrations using capillary electrophoresis is achieving high levels of precision and sensitivity to changes in lithium levels.

**Table 3:** Studies investigating electrochemical techniques for lithium therapeutic monitoring.

Reference	Type	Sensing Platform	Testing Matrix	Surface Modification/Lithium Ligand
Criscuolo et al. [14,29,30,31]	ISE, potentiometric	Metal nanostructures (SC-ISEs)	Sweat	ISM containing poly(vinyl chloride) and Li ionophore VI (6,6- Dibenzyl-1,4,8-11-tetraoxacyclotetradecane)
Sweilam et al. [17,75]	ISE, potentiometric	Cotton-fiber-based lithium sensor	ISF	Lithium sensor fabricated by dipping a cotton thread in SWCNT ink and lithium membrane solution
Lindino et al. [70]	ISE, potentiometric	Gold electrode	Serum	Conducting polymer [poly(o-methoxyaniline)]
Hanitra et al. [73,74]	ISE, potentiometric	Multi-channel electrochemical sensing	Water	ISM containing poly(vinyl chloride) and Li ionophore VI (6,6-Dibenzyl-1,4, 8-11-tetraoxacyclotetradecane)
Singh et al. [76]	ISE, voltammetric	Screen-printed sensor strips	Serum	14-crown-4 ether (6,6′-dibenzyl- 14-crown-4 ether)-based ionophore
Coldur et al. [49,71,72]	ISE, potentiometric	Potentiometric flow injection system	Serum	Solvent polyvinyl chloride (PVC) membrane
Suherman et al. [24]	ISE, voltammetric	(LiMn2O4)-modified glassy carbon electrodes (LMO-GCEs) and screen-printed electrodes (LMO-SPEs)	Saliva	Electrochemical sensing of lithium based on the galvanostatic delithiation of LMO followed by linear stripping voltammetry (LSV)
Metzger et al. [40]	ISE, potentiometric	Ag/AgCl electrodes	Serum	PVC membrane containing N,N-dlcyciohexyi-N’,N’-diiso- butyCclscyclohexane-l,2dicarboxamlde (ETH 1810)
Bertholf et al. [41]	ISE	ISEs coupled with Du Pont Na/K/Li analyzer	Serum	PVC membrane
Novell et al. [15]	ISE, potentiometric	Paper-based potentiometric cell	Blood	Polymeric membrane
Floris et al. [79]	Microchip capillary electrophoresis	Conductivity detection	Blood	N/A
Jamal et al. [36]	Capillary zone electrophoresis	Indirect UV detection	Serum and urine	N/A
Vrouwe et al. [42,77,78]	Microchip capillary electrophoresis	Conductivity detection	Blood	N/A
Kuban et al. [37]	Microchip capillary electrophoresis	Conductivity detection	Serum and urine	N/A

## 6. Discussion

The current review presents a comprehensive analysis of the investigated technologies for therapeutic monitoring of lithium levels. Several analytical methods have been described for lithium detection in different types of samples, including the routine flame emission photometry (FEP) and atomic absorption spectroscopy (AAS) techniques as well as more recent techniques such as ISEs, spectrophotometry, colorimetry, fluorometry, and capillary electrophoresis. Flame emission photometry (FEP) and atomic absorbance spectroscopy (AAS), which are the common laboratory methods for monitoring blood lithium levels, do not offer high throughput or near-patient monitoring capability, and are associated with high establishment cost and constant maintenance which makes them cumbersome for routine analysis. Research efforts have been made towards the development of automatic and lab-on-a-chip (LOC) analytical devices for lithium determination. Despite the huge expansion in recent years of lithium-sensing technologies, several challenges still exist, including poor sample collection, materials toxicity, separate sampling and analysis, and low multi-sensing capabilities [31]. Moreover, most of the developed LOC analytical devices for lithium determination are limited by constraints associated with reproducibility, and simplicity of the fabrication process [30]. The difficulty of accurately measuring lithium in biological matrices arises from the relatively high concentration of sodium, which is 100 to 300 times higher than that of lithium, at around 140 mmol/L [12]. Therefore, developing sensors for the measurement of lithium in blood requires high selectivity. Accordingly, a number of studies have investigated reagents and ionophores that exhibit high selectivity for lithium compared to sodium [71].

In order to achieve detection and quantification of lithium in different matrices, conductive polymers and ionophore-based methods have been incorporated into optical and electrochemical sensors (Table 2 and Table 3). Ionophores allow lithium detection based on Li^+^-ionophore affinity. Ionophore-based sensors, whether colorimetric, fluorescent, or electrochemical, have the greatest potential for specificity and selectivity. However, chemical ligands such as organic molecules or inorganic materials which have been investigated as lithium ligands suffer from some limitations. One of the constraints associated with employing organic chromophores for practical Li^+^ detection is their lack of solubility in aqueous media. Organic ligands are also limited by poor solubility, weak binding ability for Li, and unsatisfactory sensing performances in aqueous solutions [63]. Furthermore, alkali metal ions are known to form stable complexes with crown ethers. While these crown-ether-based devices exhibit the high selectivity that is necessary for clinical analysis of lithium, they involve cumbersome multistep procedures of fabrication [76]. There are also limitations associated with the employment of crown ethers for Li detection as the response ranges and sensitivities in relevant mediums are hard to continually improve [63]. Lastly, the chromogenic agents used for colorimetric detection of lithium are often corrosive and hazardous to ship, and highly sensitive to light and carbon dioxide in the atmosphere; the latter can result in poor precision. Such agents also require sample preparation and have extremely high pH.

Employing optical techniques for monitoring lithium levels offers numerous advantages including high accuracy, specificity, and good biocompatibility. Nevertheless, development of optical lithium sensors is associated with some challenges. For example, the use of optical lithium ligands is limited by the intrinsic properties of lithium cation such as its small size and its high charge density which can result in poor coordination ability. Therefore, the right optical lithium ligand should be identified to allow selective and efficient sensing of lithium [55,66]. Furthermore, in the development of optical lithium ligands, the response time for signal detection should be considered. The response time of small ligands used for lithium detection has been reported to vary from a few seconds to 30 min, with most of the optical sensors displaying detection after 1–10 min, because of the time needed for Li^+^ cation to coordinate with ionophore, and the necessary duration for stabilizing the detection signal [50]. Therefore, the equilibration time for Li^+^ coordination must be considered which depends on the ligand that is employed in the membrane for preparation of the optical sensor. The reversibility of ligands for lithium detection is another crucial parameter to be considered. This is because after Li^+^ detection the lithium sensor is classically non-reusable and should only be reused in the condition of a total de-coordination of lithium cation from coordination sites and the recovery of initial optical properties [50]. Ultimately, the optical lithium detection/quantification must not or must only slightly affect the studied biological system.

Lithium determination using FES, FAAS, and ISE methods has been evaluated [68]. It has been suggested that although the percentage of lithium recovery for FES and FAAS methods was high, ISE showed the most satisfactory results for recovery of lithium in pooled patient’s serum [68]. Nevertheless, it is suggested that FES remains the preferred method for lithium measurement in many laboratories. This is mainly due to the lack of standardization and validation of novel technologies which calls for a strong collaboration between academic, industrial, and medical partners. Moreover, it is demonstrated that a higher average lithium concentration for patients’ serum samples was measured by ISE and that ISE determination is easier and more precise. Nonetheless, the accuracy of ISEs may depend on other interfering factors [68]. For example, one of the main challenges associated with ISEs for lithium monitoring is the ongoing research on finding suitable ionophores with sufficiently high selectivity in respect to sodium [41,42]. In general, accurate measurement of lithium concentration by ISE requires either very high selectivity of the membrane for Li^+^ or an algorithm that will compensate for interference from sodium [41]. Furthermore, the electrochemical (EC) sensors giving a current response directly proportional to analyte concentration are preferred to potentiometric ISEs, which give a potential response that is proportional to the logarithm of analyte activity [80]. In general, ISEs have been mainly coupled with potentiometric and voltammetric detection methods which both suffer from limitations such as drifting of electrode-electrolyte interface impedance. Employment of electrical impedance spectroscopy is also an interesting but understudied approach for lithium detection. While tetrapolar electrical impedance spectroscopy offers a high degree of sensitivity to conductivity variations in samples, it lacks sensitivity towards lithium ion and must be combined with other techniques such as optical methods. Tripolar configurations, measuring the properties of functionalized electrodes, can also be investigated as they might offer a higher degree of selectivity for lithium determination. Solid-contact ion-selective electrodes (SC-ISEs) also have some limitations, including the need for calibration, limited selectivity, poor reliability, and potential drift [81,82]. Lastly, measuring compounds using capillary electrophoresis (CE) can also be challenging due to wall adsorption shifting performance, particularly over multiple runs. Therefore, different types of surface coatings have been investigated to reduce issues relating to adsorption effects [42]. Capillary electrophoresis microchips [37,77,79] also have several disadvantages, including complicated channel design and fabrication procedures and high-voltage manipulation using expensive instruments [46]. Moreover, band-broadening in CE is a general problem for samples containing a high concentration of ionic constituents as it can lead to poor resolution [77].

An important step in the development of a point-of-care (POC) device for therapeutic monitoring of lithium is matrix selection. Direct analysis of whole blood without any sample pretreatment remains one of the main challenges in blood monitoring. Therefore, combining sample treatment steps with the sensing methodologies on a single device can facilitate the development of a point-of-care device for lithium monitoring [42]. Blood-related matrices have some major limitations; namely, blood sampling is relatively invasive and may be impractical for certain patient populations. The costs associated with collecting, transporting, and processing blood samples are also significant [42]. As aforementioned, dried blood spot (DBS) and dried plasma spot (DPS) have been investigated for lithium analysis [21]. However, when using DBS and DPS to alleviate large-volume venous sampling, the influence of temperature, humidity, and sunlight exposure must be considered. Additionally, patients or caregivers must be well instructed to lower the chance of sample contamination. Several biological fluids such as sweat, saliva, and ISF have been investigated as alternative matrices to replace blood-based approaches. Nevertheless, the accumulated knowledge on drug concentration and therapeutic response dynamics needs to be established for different matrices to correlate drug levels with blood and plasma concentrations. Several studies have investigated saliva for lithium therapeutic monitoring. Although saliva can be collected easily even without patient stimulation, sensing in saliva is challenging as plaques and bacteria might attach on the sensor surface and compromise the efficacy of the system. Moreover, another limitation is the timing of saliva sampling since equilibrium of analyte transport between the blood and saliva must be present [83]. Lastly, the concentrations in saliva are strongly dependent on dietary intake. ISF, providing a medium between vasculature and cells and rich in molecules and metabolites targeted in TDM, has also been investigated for lithium monitoring. Nonetheless, one of the common challenges with ISF is the extraction of a reasonable amount of analytes for downstream analysis. Careful consideration must also be given to microneedles or under-the-skin excitation currents which are used to take out interstitial fluid, as these methods can cause irritation and discomfort in the dermis layer, especially during prolonged monitoring [31]. Sweat-based approaches for lithium TDM suffer from several issues as well. For example, the analyte concentration profiles may be location dependent as sweat glands are unevenly distributed, the sweat glands need to be stimulated by exercise or thermal heating to achieve analyte collection, and there can be high inter- and intra-individual variability. Other constraints regarding drug stability, concentration accuracy, sweat evaporation, and chance of contamination during the sampling process must also be considered. Finally, urine, which is investigated by several studies for lithium monitoring, requires sensitive analytical methods and is limited by the wide variation between and within patients.

In order to achieve point-of-care TDM in these matrices, the right sensing technology needs to be standardized, validated, and coupled with the right matrix and adequate sampling scheme and data transfer solution. Whilst numerous studies have employed different methodologies and reported the successful detection of lithium in various biological fluids, not all these methods can be translated into point-of-care devices for continuous lithium monitoring. For example, ISEs and capillary electrophoresis techniques might provide more rapid detection of lithium levels compared to the conventional methods in laboratory settings. However, their employment in point-of-care analysis still suffers from some limitations such as the need for high-voltage input. Furthermore, continuous monitoring of lithium levels requires high levels of biocompatibility which is often difficult to achieve with ISEs. Optical methods, on the other hand, offer a good level of biocompatibility for continuous monitoring of lithium levels. However, in the development of optical sensors, suitable lithium ligands must be employed to ensure sensitive and efficient lithium detection. Lastly, the right biological fluid should be selected to allow minimally invasive and point-of-care monitoring of lithium. The interstitial fluid, namely, is an accessible and reproducible matrix which seems to be the most suitable human body fluid for minimally invasive detection of lithium. Overall, many of the developed technologies for the monitoring of analytes cannot easily be translated into point-of-care (POC) devices as they do not lend themselves easily to operation by untrained personnel. This is because they generally involve manipulation of the sample or application of high-voltage electrodes. Therefore, the sensing technology needs to be decentralized and simple, fast, and economical of use in order to make the therapeutic monitoring of lithium feasible.

## 7. Concluding Remarks

Efforts have been made to develop minimally invasive point-of-care lithium-monitoring devices utilizing different matrices. Although such devices and methodologies show promise, the constraints associated with cost and simplicity of their instrumentation are yet to be overcome. Development of a minimally invasive lithium-monitoring method will be a major advance in lithium TDM as it will allow patients and non-medically-trained personnel to measure lithium levels and ensure patients are receiving the optimum dose. Furthermore, monitoring of drugs with a narrow therapeutic range at home using wearable sensors would help to reduce the burden on patients and health professionals associated with attendance of clinical settings and facilitate improved therapeutic monitoring. Moreover, improved rates of lithium treatment monitoring will majorly reduce the chances of adverse events. Therefore, development of facile and minimally invasive approaches to providing lithium measurements in real time will be highly desired.

## Figures and Tables

**Table 1:** Advantages and limitations of different biological fluids for therapeutic monitoring of lithium.

Biological Fluid	Advantages	Limitations
Blood	Provides accurate measurements	Relative invasiveness, cost, and impracticalities
Sweat	Non-invasiveness	Sample collection requires stimulating sweat glands, presence of potential contaminants
Saliva	Accessibility, non-invasiveness	Drug instability, presence of potential contaminants, and lack of phase II metabolites
Interstitial fluid (ISF)	Good correlation with venous blood, suitable for continuous monitoring, good reproducibility, minimally invasive	Low volume, sample evaporation
Dried blood/plasma spots	Small collection volume, minimal discomfort, and easy sample collection	Accuracy of measurement and reproducibility
Urine	High concentrations of many drugs and metabolites in urine, non-invasiveness	Unsatisfactory accuracy, the need for pretreatment, and wide variations between patients
